# Controlled Release of Granulocyte Colony-Stimulating Factor Enhances Osteoconductive and Biodegradable Properties of Beta-Tricalcium Phosphate in a Rat Calvarial Defect Model

**DOI:** 10.1155/2014/134521

**Published:** 2014-04-14

**Authors:** Tomohiro Minagawa, Yasuhiko Tabata, Akihiko Oyama, Hiroshi Furukawa, Takeshi Yamao, Yuhei Yamamoto

**Affiliations:** ^1^Department of Plastic and Reconstructive Surgery, Hokkaido University Graduate School of Medicine, Sapporo, Hokkaido 060-8638, Japan; ^2^Department of Plastic and Reconstructive Surgery, Otaru Kyokai Hospital, Suminoe-1-6-15, Otaru, Hokkaido 047-8510, Japan; ^3^Department of Biomaterials, Field of Tissue Engineering, Institute for Frontier Medical Sciences, Kyoto University, Kyoto 606-8507, Japan

## Abstract

Autologous bone grafts remain the gold standard for the treatment of congenital craniofacial disorders; however, there are potential problems including donor site morbidity and limitations to the amount of bone that can be harvested. Recent studies suggest that granulocyte colony-stimulating factor (G-CSF) promotes fracture healing or osteogenesis. The purpose of the present study was to investigate whether topically applied G-CSF can stimulate the osteoconductive properties of beta-tricalcium phosphate (**β**-TCP) in a rat calvarial defect model. A total of 27 calvarial defects 5 mm in diameter were randomly divided into nine groups, which were treated with various combinations of a **β**-TCP disc and G-CSF in solution form or controlled release system using gelatin hydrogel. Histologic and histomorphometric analyses were performed at eight weeks postoperatively. The controlled release of low-dose (1 **μ**g and 5 **μ**g) G-CSF significantly enhanced new bone formation when combined with a **β**-TCP disc. Moreover, administration of 5 **μ**g G-CSF using a controlled release system significantly promoted the biodegradable properties of **β**-TCP. In conclusion, the controlled release of 5 **μ**g G-CSF significantly enhanced the osteoconductive and biodegradable properties of **β**-TCP. The combination of G-CSF slow-release and **β**-TCP is a novel and promising approach for treating pediatric craniofacial bone defects.

## 1. Introduction

Autologous bone grafts remain the gold standard for the treatment of congenital craniofacial bone disorders, such as alveolar cleft [1–8]. However, autologous bone grafts have potential problems, which include donor site morbidity and limitations to the amount of bone that can be harvested [9–13]. Porous beta-tricalcium phosphate (*β*-TCP), which is now commercially available, is known for its osteoconductive and biodegradable properties. However, its use as a replacement for autologous bone grafts remains controversial [14–18].

According to recent studies, various growth factors exhibit osteogenic properties [8, 19–23], such as bone morphogenetic protein 2 (BMP-2) [24–36], basic fibroblast growth factor (b-FGF) [37–42], platelet derived growth factor (PDGF) [43–46], transforming growth factor-beta 1 (TGF-*β*1) [47–50], and vascular endothelial growth factor (VEGF) [51–54]. In general, growth factors administered in solution form are readily diffused or degraded* in vivo *[26, 50, 55, 56]. Thus, their enhanced and prolonged bioactivity at the target site is necessary to reduce bolus dosage, especially in pediatric patients. In addition, growth factors must be administered in combination with carrier materials.

Recent studies suggest that granulocyte colony-stimulating factor (G-CSF) promotes fracture healing or osteogenesis [57–60]. Because G-CSF is an essential drug most frequently used to treat neutropenia secondary to chemotherapy, it is widely administered not only to adults but also to pediatric patients [61–69]. Accordingly, its biosafety is well established through extensive use in clinical contexts compared to other growth factors.

Commercially available *β*-TCP (Superpore, PENTAX, Tokyo, Japan) was used in the present study as an osteoconductive scaffold and space-maintaining material. To investigate the bone regenerative properties of G-CSF, topical supplementation either in solution or in sustained release form with a gelatin hydrogel system was performed. The purpose of this study was to investigate whether G-CSF with or without a controlled release system stimulates bone regeneration in combination with *β*-TCP using a rat calvarial defect model [70–73].

## 2. Materials and Methods

### 2.1. Study Design and Ethics

The present study was approved by the institutional committee of animal experiments at Hokkaido University (Institutional Animal Care and Use Committee Protocol number 12-0017). Fourteen Wistar rats (male, 13 weeks old; weight, 250–350 g) were purchased from Sankyo Labo Service Corporation (Tokyo, Japan). A total of 27 calvarial defects were randomly divided into nine treatment groups, with a total of three defects per treatment group. In solution-based treatment groups, defects were filled with a *β*-TCP disc containing normal saline alone (group A, control) or 1 (group B), 5 (group C), or 20 *μ*g of G-CSF (group D). In controlled release groups, defects were filled with a *β*-TCP disk with an overlaid gelatin hydrogel sheet incorporating normal saline alone (group E) or, 1 (group F), 5 (group G), or 20 *μ*g G-CSF (group H). The remaining defects were left empty to measure spontaneous healing (group I) (Table 1).

### 2.2. Preparation of *β*-TCP and Gelatin Hydrogel Incorporating G-CSF

Commercially available porous *β*-TCP blocks (Superpore) were kindly supplied by PENTAX (Tokyo, Japan). Blocks were cut into discs 5 mm in diameter and 1 mm thick using a fine surgical saw and round bur.

Gelatin hydrogels were prepared by glutaraldehyde crosslinking of acidic gelatin as previously described [37]. Briefly, a mixed acidic gelatin-glutaraldehyde aqueous solution was cast into a polypropylene dish (80 × 80 mm^2^) and maintained at 4°C for 12 hours. Hydrogel sheets were placed in a 100 mM glycine aqueous solution at 37°C. Discs were freeze-dried and sterilized with ethylene oxide gas. The water content of gelatin hydrogels (weight ratio of water present in hydrogel to wet hydrogel) was 95 wt%. Gelatin hydrogels were designed so that degradation would be complete in approximately two weeks under* in vivo* conditions [29, 39, 49, 74].

Hydrogel sheets were cut into discs 5 mm in diameter and 1 mm thick. Human recombinant G-CSF was kindly supplied by KYOWA KIRIN Co. (Tokyo, Japan). To prepare gelatin hydrogels incorporating G-CSF, 20 *μ*L of normal saline solution containing 1, 5, or 20 *μ*g G-CSF was dropped onto freeze-dried hydrogel discs and left at 4°C overnight. Similarly, 20 *μ*L of G-CSF-free normal saline was dropped onto a freeze-dried hydrogel to obtain G-CSF empty hydrogels.

### 2.3. Surgical Procedures

**Figure 1:**
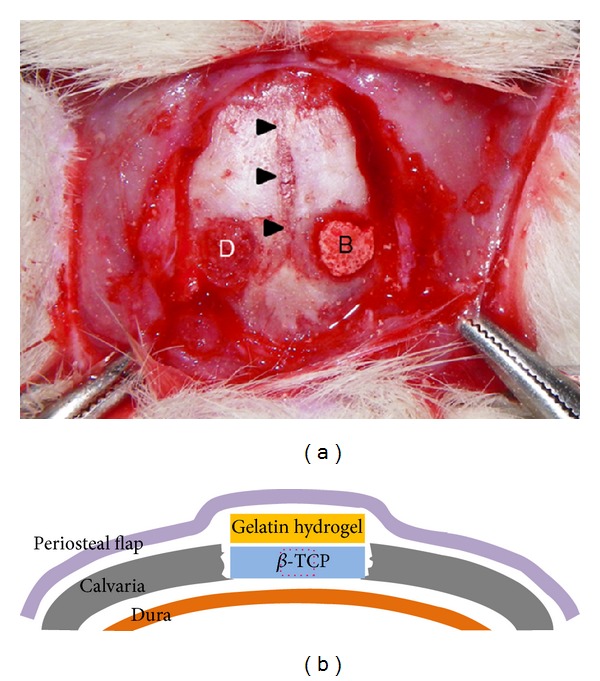
Surgical images. (a) Intraoperative view of rat calvaria. Two 5 mm diameter calvarial defects were created on each side of the sagittal suture (arrow heads). The defects were filled with a beta-tricalcium phosphate disc (B) or left untreated with exposed dura (D). (b) Schematic of a cross section of the calvarial defect. The dotted square in *β*-TCP indicates the most-central area where histomorphometric analysis was conducted.

Animals were anesthetized by intraperitoneal administration of pentobarbital sodium (50 mg/kg). Surgical areas were shaved and disinfected with povidone-iodine. Subsequently, a skin incision was made and subperiosteal dissection was performed under a surgical microscope to raise the periosteal flaps. A bone defect 5 mm in diameter was then prepared on each side lateral to the sagittal suture using a fine surgical bur under copious sterile saline irrigation. Defects were filled with bone substitutes according to the groups described above (Table 1 and Figure 1). Periosteal flaps were repositioned using a 4-0 nylon suture, and the skin was closed with a running 4-0 nylon suture. Finally, animals were euthanized by anesthetic overdose eight weeks after surgery.

### 2.4. Histological Processing

Specimens were prepared for decalcified sectioning by immersing them in 10% ethylenediaminetetraacetic acid (EDTA) for four weeks. Decalcified specimens were dehydrated in ascending grades of ethanol and embedded in paraffin wax. Embedded samples were then sectioned into 3 *μ*m slices parallel to the sagittal suture across the center of each calvarial defect using a microtome (LEICA, SM2000R). Hematoxylin and eosin (HE) staining was used for histological analysis and aniline blue staining was used for histomorphometric analysis.

### 2.5. Histologic and Histomorphometric Analysis

Each specimen was examined under a light microscope and digital photographs were obtained for histological evaluation of a region corresponding to the center of the calvarial defect (Figure 1(b)). Images of HE staining were used for conventional histological analysis. High magnification images with aniline blue staining (1.001 mm^2^ or 1360 × 1024 pixels) of the most-central area of the defect were quantified to measure the percentage of newly formed bone and remaining bioceramics using imaging software (Adobe Photoshop CS5) [75]. All histomorphometric evaluations were conducted by a researcher blinded to the groupings.

### 2.6. Statistical Analysis

Statistical analysis was performed using Kruskal-Wallis one-way analysis of variance (ANOVA). Data between groups were further analyzed using a Tukey-Kramer multiple comparisons test. *P* < 0.05 was considered statistically significant. Experimental results were expressed as mean ± standard deviation (SD).

## 3. Results

### 3.1. Histological Findings

**Figure 2:**
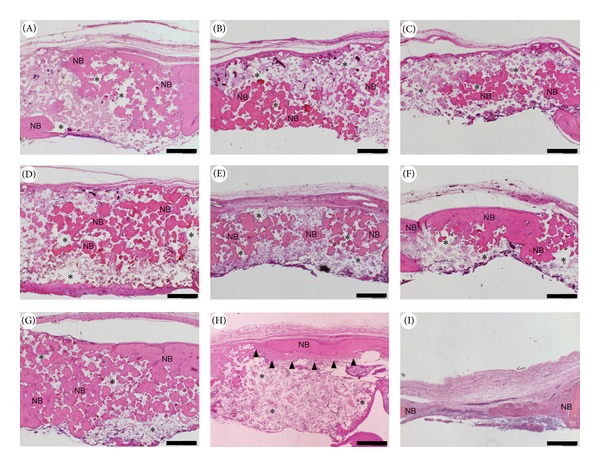
Low magnification images of hematoxylin and eosin (HE) staining. (a) *β*-TCP alone; (b) 1 *μ*g G-CSF in solution form; (c) 5 *μ*g G-CSF in solution form; (d) 20 *μ*g G-CSF in solution form; (e) free gelatin hydrogel; (f) 1 *μ*g G-CSF with gelatin hydrogel; (g) 5 *μ*g G-CSF with gelatin hydrogel; (h) 20 *μ*g G-CSF with gelatin hydrogel; and (i) untreated defect. Note the dense fibrous tissue surrounding focal bone formation in group H (arrow heads). Original magnification 40x; scale bar = 500 *μ*m. NB: newly formed bone; *remaining *β*-TCP.

**Figure 3:**
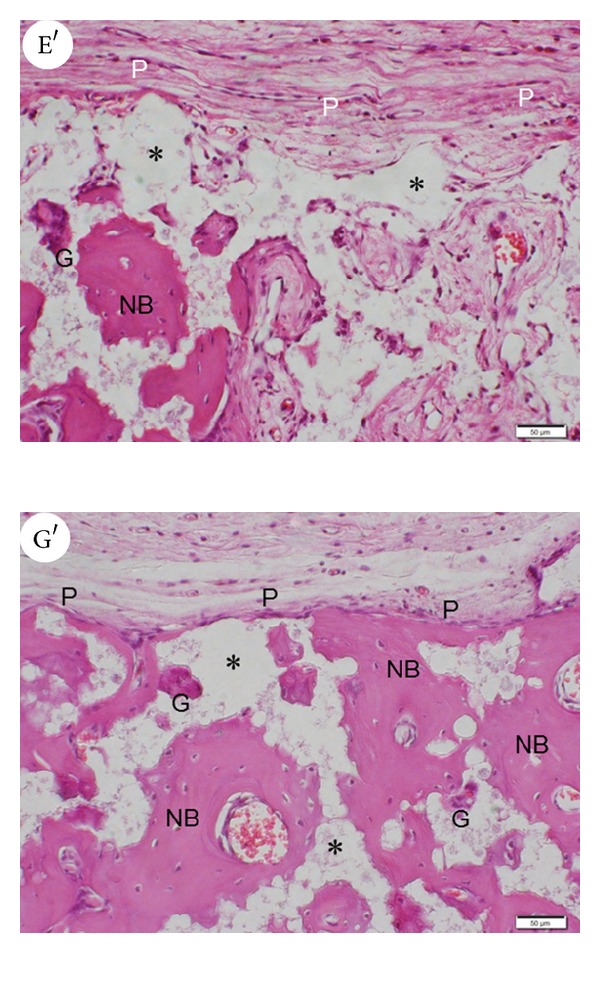
High magnification images of hematoxylin and eosin staining (E′) group E and (G′) group G. Note the obvious fibrous tissue and vessel formation in group E. In group G, newly formed bone is observed immediately below the periosteal layer and multinuclear giant cells are present. Original magnification 200x; scale bar = 50 *μ*m. G: multinuclear giant cell; NB: newly formed bone; P: periosteal flap; *remaining *β*-TCP.

Figure 2 shows low magnification images of decalcified specimens stained with HE along the midline of each calvarial defect. No remaining gelatin hydrogel or surgical site infections were observed. In group A (control group), newly formed trabecular bone was observed focally but failed to occupy the entire defect. In groups B–D (solution-based treatment groups) and in group E (G-CSF-free gelatin hydrogel group), newly formed trabecular bone was observable but failed to fill the defect. In group F (1 *μ*g G-CSF gelatin hydrogel group), newly formed bone tissue nearly bridged the calvarial gap, whereas residual *β*-TCP was also present. In group G (5 *μ*g G-CSF gelatin hydrogel group), most of the defect was occupied with newly formed bone tissue; moreover, sparse residual *β*-TCP was observed. In contrast, group H (20 *μ*g G-CSF gelatin hydrogel group) showed focal formation of new bone surrounded by fibrous connective tissue at the superficial area of the defect with the presence of remaining biomaterials. In group I (untreated defect group), the defect was filled with fibrous connective tissue with hardly any newly formed bone.  Figure 3 shows higher magnification images of groups E (E′) and G (G′). In group G, newly formed bone was observed immediately below the periosteal flap and multinuclear giant cells were detected around the newly formed bone. In contrast, in group E, the formation of fibrous tissue and blood vessels was significant compared with newly formed bone in the subperiosteal region.

### 3.2. Histomorphometric Evaluation

**Figure 4:**
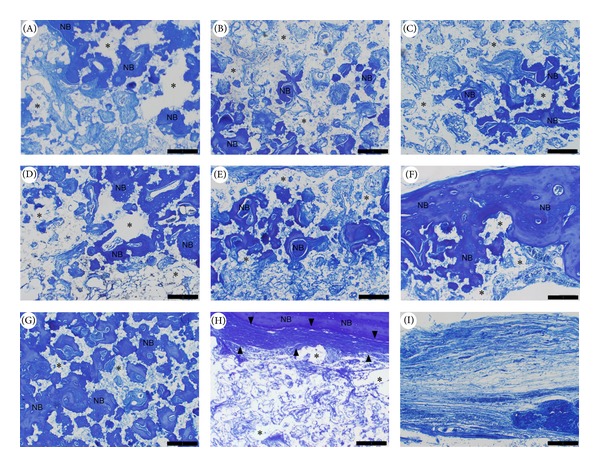
High magnification images of aniline blue staining. Capital letters A–I correspond to the treatment groups. Newly formed bone (NB) appears as a homogenous dark blue area, which includes osteocytes. The remaining *β*-TCP shows a homogenous white area (asterisk). Bony bridging was nearly complete in groups F and G. Less residual *β*-TCP was seen in group G. Original magnification 100x; scale bar = 200 *μ*m.

**Figure 5:**
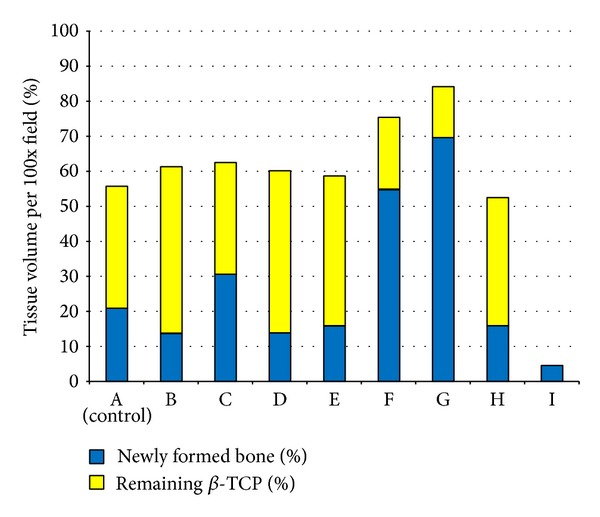
Percentage of newly formed bone and remaining *β*-TCP eight weeks after surgery. Values are shown as mean. Capital letters A–I correspond to treatment groups. Note that newly formed bone occupies more than 50% of the central area of the defect in groups F and G. In addition, the percentage of newly formed bone exceeds that of remaining *β*-TCP.

Figure 4 shows high magnification images of aniline blue staining in which matured bone tissue exhibits homogeneous dark blue and entrapped osteocytes. Residual *β*-TCP was observed as homogeneous white particles.  Figure 5 shows the percentage of newly formed bone and remaining *β*-TCP per high-powered field. In groups A, B, C, D, E, and H, defects had a tendency to be occupied by more remaining *β*-TCP compared to newly formed bone tissue. In group A (control), the percentages of newly formed bone and remaining *β*-TCP were 20.77% ± 25.44% and 35.01% ± 2.01%, respectively. In contrast, in groups F and G (1 *μ*g and 5 *μ*g G-CSF gelatin hydrogel groups), the percentage of newly formed bone (54.84% ± 9.46% and 69.53% ± 5.35% for groups F and G, resp.) conspicuously exceeded values of remaining *β*-TCP (20.47% ± 2.89% and 14.76% ± 7.36% for groups F and G, resp.).

**Figure 6:**
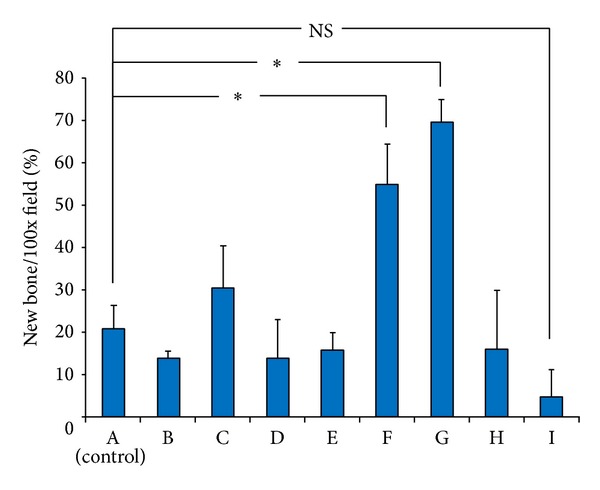
Percentage of newly formed bone in the high magnification field. Values are shown as mean ± standard deviation (SD, *P < 0.01). The values are significantly higher in groups F and G (1 and 5 *μ*g G-CSF with gelatin hydrogel, resp.) compared to group A (*β*-TCP alone).

Figure 6 shows the percentage of newly formed bone after statistical analysis. The values were significantly higher in groups F and G compared to the control group (*P* < 0.01). There was no significant difference between groups A (control) and I (empty defect). Values corresponding to groups B, C, D, E, and H showed no significant difference compared to that of group A.

**Figure 7:**
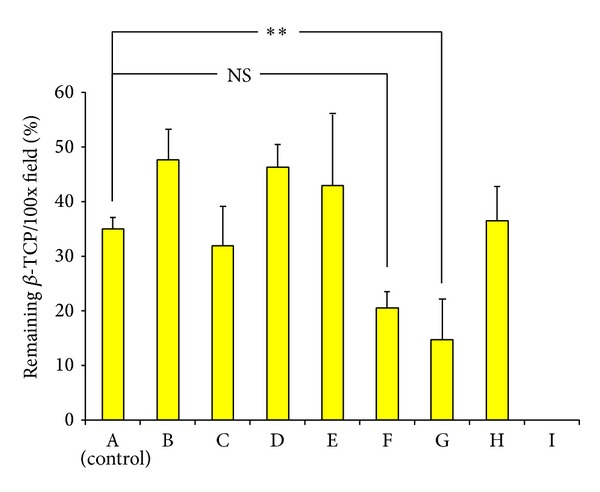
Percentage of remaining *β*-TCP in the high magnification field. Values are shown as mean ± SD (**P < 0.05). The percentage in group G (5 *μ*g G-CSF with gelatin hydrogel) is significantly lower compared to group A. This result highlights the biodegradable properties of *β*-TCP.

Figure 7 shows the percentage of remaining *β*-TCP, which was used to evaluate biodegradability* in vivo*. There was no significant difference between groups A, B, C, D, E, F, and H. In contrast, only in group G (5 *μ*g G-CSF gelatin hydrogel group) the percentage was significantly lower compared to group A (14.76% ± 7.36% versus 33.53% ± 0.80%, *P* < 0.05). This result indicated a prominent enhancement of the biodegradable properties of *β*-TCP, which was further accelerated by 5 *μ*g G-CSF in sustained release form.

## 4. Discussion

In the present study, we demonstrated that the controlled release of low-dose (1 *μ*g and 5 *μ*g) G-CSF significantly enhanced bone regeneration when combined with a *β*-TCP disc. Moreover, administration of 5 *μ*g G-CSF using a controlled release system significantly promoted the biodegradable properties of *β*-TCP. According to our results, this tissue-engineering approach combining *β*-TCP and the sustained release of G-CSF is potentially feasible and promising for clinical use. To our knowledge, this is the first report which demonstrates the bone regeneration properties of G-CSF at membranous ossification sites. Because systemic administration of 5–10 *μ*g G-CSF/kg/day is commonly used for pediatric malignancies [64, 66–68], the results shown here indicate that notably low doses of G-CSF (1–5 *μ*g/defect/2 weeks) with controlled release can promote osteogenesis.

In this study, we used *β*-TCP as an osteoconductive scaffold and space-maintaining material [76]. In the present study, the left untreated defect group showed thin connective tissue formation with minimal bone regeneration. Although the control group (*β*-TCP alone) showed a greater tendency for bone formation compared to the untreated defect group, there were no significant differences between the groups. Furthermore, the defect in the control group had more residual *β*-TCP than newly formed bone tissue. These results suggest that *β*-TCP alone implantation is not sufficient to fill the defect with regenerated bone in the craniofacial region. Some experimental studies have confirmed the osteoconductive properties of *β*-TCP, which were comparable to autologous bone grafts [15, 17, 70]. However, other groups have emphasized versatility by combining *β*-TCP with autologous bone fragments [16, 77–79], growth factors [45, 46, 80–84], simvastatin [71], or stem cells [18, 85, 86] in both experimental and clinical studies.

Interestingly, Ishida et al. reported that topical application of G-CSF had bone regenerative properties via neovascularization and osteogenesis [59]. That study revealed a significant increase in CD34^+^ cells—an endothelial and hematopoietic progenitor-enriched cell population—in capillaries corresponding to the bone defect site. These findings suggested that CD34^+^ cells were important promoters of neovascularization. The study also showed that G-CSF was responsible for mobilizing osteoblasts to the bone defect site. In addition, recent studies demonstrated the promotion of fracture healing by CD34^+^ cells [58, 87–89]. Kuroda et al. reported the first successful clinical case of a tibial nonunion treated with topically applied G-CSF-mobilized CD34^+^ cells [60]. Some reports have shown that CD34^+^ cells play an important role in releasing angiogenic factors, including vascular endothelial growth factor (VEGF), hepatocyte growth factor (HGF), and fibroblast growth factor 2 (FGF2) [58, 90, 91]. Moreover, the differentiation capacity of CD34^+^ cells into osteoblasts has been shown in previous reports [59, 92]. In the present study, the controlled release G-CSF groups showed more newly formed bone immediately below the periosteum compared to the other groups. On the other hand, Rojbani et al. reported that osteoprogenitor cells differentiate from the dura mater [71]. Presumably, the sustained release of G-CSF may stimulate periosteal cells along an osteogenic lineage, resulting in enhanced bone formation.

In the present study, we used gelatin hydrogel as a sustained release carrier of G-CSF. Various growth factors have been shown to have bone regenerative properties, such as bone morphogenetic proteins (BMPs) [24–36], b-FGF [37–42, 93], PDGF [43–46], TGF-*β*1 [47–50], and VEGF [51–54]. BMP-2 has the strongest osteoinductive activity in promoting ectopic bone regeneration [26, 29] and has been approved by the Food and Drug Administration for use in orthopedics and oral surgery [30, 34, 35]. In general, growth factors administered in solution form are easily diffused or degraded prior to achieving full bioactivity [26, 50, 55, 56]. Therefore, commercially available BMP-2 in combination with a collagen sponge kit must contain milligram amounts of the growth factor (1.5 mg/mL) [30, 34, 35]. Potential risk for local inflammatory responses should be taken into consideration after topical application [35]. In order to reduce bolus dosage, enhanced and prolonged bioactivity of growth factors at the targeting site is necessary. One of the practical ways to control the* in vivo* release of growth factors is to use gelatin hydrogel, in which the growth factor is physicochemically immobilized and subsequently released in proportion to hydrogel degradation [74, 94]. In the present study, the water content of gelatin hydrogels (weight ratio of water present in hydrogel to wet hydrogel) was 95 wt%. The hydrogels were designed so that degradation would be complete in approximately 2 weeks under* in vivo* conditions [29, 39, 49, 50, 74]. Gelatin is commercially available and its biosafety is well established through its long clinical use as a plasma expander and drug ingredient.

In the present study, the controlled release of 5 *μ*g G-CSF (group G) significantly promoted the osteoconductive properties and biodegradability of *β*-TCP. Improved biodegradability compared to hydroxyapatite is a major characteristic of porous *β*-TCP [71, 95–97]. Biodegradability is generally thought to occur in harmony with bone remodeling, in which *β*-TCP allows tissue fluid dissolution and absorption by osteoclasts* in vivo *[72, 95]. Brouard et al. reported that G-CSF increased both osteoclast activity and bone resorption in the bone marrow, triggering an increase in the number of mesenchymal precursor cells in the bone marrow using a mouse model [98]. In another study, PDGF modified *β*-TCP resorption, although the underlying mechanism was not provided [46]. Some studies have shown that BMP-2 does not facilitate *β*-TCP resorption [70, 99]. In group G of the present study, multinuclear giant cells were observed around newly formed bone immediately below the periosteum. We can speculate from the results that the controlled release of 5 *μ*g G-CSF may stimulate the mobilization and differentiation of mesenchymal precursor cells in the periosteum as well as osteoclast activation. In contrast, group H (20 *μ*g G-CSF gelatin hydrogel group) showed less new bone formation and *β*-TCP resorption. This might be explained by the multidifferentiation potential of G-CSF-mobilized progenitor cells, which is consistent with previously published reports [92, 100–102]. Interestingly, Ishida et al. stated that topical application of 50 *μ*g G-CSF did not induce bone regeneration according to preliminary data [59]. Moreover, some reports have shown that sustained release of G-CSF enhances tendon-bone integration with significantly more formation of Sharpey's fibers and microvessels [103]. These results led us to speculate that a prolonged high concentration of topical G-CSF drives progenitor cells toward fibrous tissue formation rather than osteogenesis. Therefore, sustaining relatively low concentrations of topical G-CSF can play an important role in inducing balanced bone regeneration and *β*-TCP resorption. Our findings suggest an optimal dose of 5 *μ*g per defect for controlled release of G-CSF, which is consistent with previously published reports [59, 103].

There are several limitations in this study that must be noted. First, the study was designed using small animals and a limited number per experimental group. Second, although some reports accept the calvarial defect rat model [70–73], the decortication procedure may not fully reflect clinical situations of congenital craniofacial anomalies [104], since some evidence suggests that fractures mobilize CD34^+^ cells from the bone marrow into the peripheral blood [88, 89]. Third, we used histomorphometric analysis to characterize newly formed bone and biodegradation of *β*-TCP; however, we did not identify CD34^+^ cells or evaluate the activity of osteogenic cells at the bone defect site. Future studies should incorporate experimental models without decortication, larger animals, and immunohistochemical analysis.

In conclusion, controlled release of 5 *μ*g G-CSF using a gelatin hydrogel system significantly enhances the osteoconductive and biodegradable properties of porous *β*-TCP. The present results indicate that the combination of G-CSF slow-release and *β*-TCP is feasible and promising for the treatment of congenital craniofacial bone defects.

## Figures and Tables

**Table 1:** List of the experimental groups.

*β*-TCP	Types of G-CSF administration	Rat grouping (A–I)/			
		G-CSF dose (*μ*g/defect)			
		0	1	5	20
+	Solution form	A	—	—	—
+	Solution form	—	B	—	—
+	Solution form	—	—	C	—
+	Solution form	—	—	—	D
+	Controlled release using gelatin hydrogel	E	—	—	—
+	Controlled release using gelatin hydrogel	—	F	—	—
+	Controlled release using gelatin hydrogel	—	—	G	—
+	Controlled release using gelatin hydrogel	—	—	—	H
None	Solution form	I	—	—	—

## References

[1] Boyne PJ (1974). Use of marrow cancellous bone grafts in maxillary alveolar and palatal clefts. *Journal of Dental Research*.

[2] Abyholm FE, Bergland O, Semb G (1981). Secondary bone grafting of alveolar clefts. A surgical/orthodontic treatment enabling a non-prosthodontic rehabilitation in cleft lip and palate patients. *Scandinavian Journal of Plastic and Reconstructive Surgery*.

[3] Jackson IT, Scheker LR, Vandervord JG, McLennan JG (1981). Bone marrow grafting in the secondary closure of alveolar-palatal defects in children. *British Journal of Plastic Surgery*.

[4] Enemark H, Sindet-Pedersen S, Bundgaard M (1987). Long-term results after secondary bone grafting of alveolar clefts. *Journal of Oral and Maxillofacial Surgery*.

[5] Cohen M, Polley JW, Figueroa AA (1993). Secondary (intermediate) alveolar bone grafting. *Clinics in Plastic Surgery*.

[6] LaRossa D, Buchman S, Rothkopf DM, Mayro R, Randall P, Wolfe SA (1995). A comparison of iliac and cranial bone in secondary grafting of alveolar clefts. *Plastic and Reconstructive Surgery*.

[7] Bajaj AK, Wongworawat AA, Punjabi A (2003). Management of alveolar clefts. *The Journal of Craniofacial Surgery*.

[8] van Hout WMMT, van der Molen ABM, Breugem CC, Koole R, van Cann EM (2011). Reconstruction of the alveolar cleft: can growth factor-aided tissue engineering replace autologous bone grafting? A literature review and systematic review of results obtained with bone morphogenetic protein-2. *Clinical Oral Investigations*.

[9] Sadove AM, Nelson CL, Eppley BL, Nguyen B (1990). An evaluation of calvarial and iliac donor sites in alveolar cleft grafting. *Cleft Palate Journal*.

[10] Dawson KH, Egbert MA, Myall RWT (1996). Pain following iliac crest bone grafting of alveolar clefts. *Journal of Cranio-Maxillo-Facial Surgery*.

[11] Swan MC, Goodacre TEE (2006). Morbidity at the iliac crest donor site following bone grafting of the cleft alveolus. *British Journal of Oral and Maxillofacial Surgery*.

[12] Rawashdeh MA, Telfah H (2008). Secondary alveolar bone grafting: the dilemma of donor site selection and morbidity. *British Journal of Oral and Maxillofacial Surgery*.

[13] Baqain ZH, Anabtawi M, Karaky AA, Malkawi Z (2009). Morbidity from anterior Iliac crest bone harvesting for secondary alveolar bone grafting: an outcome assessment study. *Journal of Oral and Maxillofacial Surgery*.

[14] Szabó G, Huys L, Coulthard P (2005). A prospective multicenter randomized clinical trial of autogenous bone versus *β*-tricalcium phosphate graft alone for bilateral sinus elevation: histologic and histomorphometric evaluation. *International Journal of Oral and Maxillofacial Implants*.

[15] Zijderveld SA, Zerbo IR, van den Bergh JPA, Schulten EAJM, Ten Bruggenkate CM (2005). Maxillary sinus floor augmentation using a *β*-tricalcium phosphate (Cerasorb) alone compared to autogenous bone grafts. *International Journal of Oral and Maxillofacial Implants*.

[16] Dai L-Y, Jiang L-S (2008). Single-level instrumented posterolateral fusion of lumbar spine with *β*-tricalcium phosphate versus autograft: a prospective, randomized study with 3-year follow-up. *Spine*.

[17] de Ruiter A, Meijer G, Dormaar T (2011). *β*-TCP versus autologous bone for repair of alveolar clefts in a goat model. *Cleft Palate-Craniofacial Journal*.

[18] Zhang D, Chu F, Yang Y (2011). Orthodontic tooth movement in alveolar cleft repaired with a tissue engineering bone: an experimental study in dogs. *Tissue Engineering A*.

[19] Jadlowiec JA, Celil AB, Hollinger JO (2003). Bone tissue engineering: recent advances and promising therapeutic agents. *Expert Opinion on Biological Therapy*.

[20] Salgado AJ, Coutinho OP, Reis RL (2004). Bone tissue engineering: state of the art and future trends. *Macromolecular Bioscience*.

[21] Moreau JL, Caccamese JF, Coletti DP, Sauk JJ, Fisher JP (2007). Tissue engineering solutions for cleft palates. *Journal of Oral and Maxillofacial Surgery*.

[22] Alvarez P, Hee CK, Solchaga L (2012). Growth factors and craniofacial surgery. *Journal of Craniofacial Surgery*.

[23] Behr B, Sorkin M, Lehnhardt M, Renda A, Longaker MT, Quarto N (2012). A comparative analysis of the osteogenic effects of BMP-2, FGF-2, and VEGFA in a calvarial defect model. *Tissue Engineering A*.

[24] Mayer M, Hollinger J, Ron E, Wozney J (1996). Maxillary alveolar cleft repair in dogs using recombinant human bone morphogenetic protein-2 and a polymer carrier. *Plastic and Reconstructive Surgery*.

[25] Hong L, Tabata Y, Yamamoto M (1998). Comparison of bone regeneration in a rabbit skull defect by recombinant human BMP-2 incorporated in biodegradable hydrogel and in solution. *Journal of Biomaterials Science, Polymer Edition*.

[26] Yamamoto M, Tabata Y, Ikada Y (1998). Ectopic bone formation induced by biodegradable hydrogels incorporating bone morphogenetic protein. *Journal of Biomaterials Science, Polymer Edition*.

[27] Higuchi T, Kinoshita A, Takahashi K, Oda S, Ishikawa I (1999). Bone regeneration by recombinant human bone morphogenetic protein-2 in rat mandibular defects. An experimental model of defect filling. *Journal of Periodontology*.

[28] Boyne PJ (2001). Application of bone morphogenetic proteins in the treatment of clinical oral and maxillofacial osseous defects. *The Journal of Bone & Joint Surgery A*.

[29] Yamamoto M, Takahashi Y, Tabata Y (2003). Controlled release by biodegradable hydrogels enhances the ectopic bone formation of bone morphogenetic protein. *Biomaterials*.

[30] Chin M, Ng T, Tom WK, Carstens M (2005). Repair of alveolar clefts with recombinant human bone morphogenetic protein (rhBMP-2) in patients with clefts. *Journal of Craniofacial Surgery*.

[31] Yamamoto M, Takahashi Y, Tabata Y (2006). Enhanced bone regeneration at a segmental bone defect by controlled release of bone morphogenetic protein-2 from a biodegradable hydrogel. *Tissue Engineering*.

[32] Herford AS, Boyne PJ, Rawson R, Williams RP (2007). Bone morphogenetic protein-induced repair of the premaxillary cleft. *Journal of Oral and Maxillofacial Surgery*.

[33] Herford AS, Boyne PJ, Williams RP (2007). Clinical applications of rhBMP-2 in maxillofacial surgery. *Journal of the California Dental Association*.

[34] Dickinson BP, Ashley RK, Wasson KL (2008). Reduced morbidity and improved healing with bone morphogenic protein-2 in older patients with alveolar cleft defects. *Plastic and Reconstructive Surgery*.

[35] Alonso N, Tanikawa DYS, Freitas RDS, Canan L, Ozawa TO, Rocha DL (2010). Evaluation of maxillary alveolar reconstruction using a resorbable collagen sponge with recombinant human bone morphogenetic protein-2 in cleft lip and palate patients. *Tissue Engineering C: Methods*.

[36] Asamura S, Mochizuki Y, Yamamoto M, Tabata Y, Isogai N (2010). Bone regeneration using a bone morphogenetic protein-2 saturated slow-release gelatin hydrogel sheet: evaluation in a canine orbital floor fracture model. *Annals of Plastic Surgery*.

[37] Yamada K, Tabata Y, Yamamoto K (1997). Potential efficacy of basic fibroblast growth factor incorporated in biodegradable hydrogels for skull bone regeneration. *Journal of Neurosurgery*.

[38] Tabata Y, Yamada K, Miyamoto S (1998). Bone regeneration by basic fibroblast growth factor complexed with biodegradable hydrogels. *Biomaterials*.

[39] Tabata Y, Yamada K, Hong L, Miyamoto S, Hashimoto N, Ikada Y (1999). Skull bone regeneration in primates in response to basic fibroblast growth factor. *Journal of Neurosurgery*.

[40] Iwakura A, Tabata Y, Miyao M (2000). Novel method to enhance sternal healing after harvesting bilateral internal thoracic arteries with use of basic fibroblast growth factor. *Circulation*.

[41] Iwakura A, Tabata Y, Tamura N (2001). Gelatin sheet incorporating basic fibroblast growth factor enhances healing of devascularized sternum in diabetic rats. *Circulation*.

[42] Hayashi K, Kubo T, Doi K, Tabata Y, Akagawa Y (2007). Development of new drug delivery system for implant bone augmentation using a basic fibroblast growth factor-gelatin hydrogel complex. *Dental Materials Journal*.

[43] Arm DM, Tencer AF, Bain SD, Celino D (1996). Effect of controlled release of platelet-derived growth factor from a porous hydroxyapatite implant on bone ingrowth. *Biomaterials*.

[44] Pellegrini G, Seol YJ, Gruber R, Giannobile WV (2009). Pre-clinical models for oral and periodontal reconstructive therapies. *Journal of Dental Research*.

[45] Young CS, Ladd PA, Browning CF (2009). Release, biological potency, and biochemical integrity of recombinant human platelet-derived growth factor-BB (rhPDGF-BB) combined with AugmentTM Bone Graft or GEM 21S beta-tricalcium phosphate (*β*-TCP). *Journal of Controlled Release*.

[46] Choo T, Marino V, Bartold PM (2013). Effect of PDGF-BB and beta-tricalcium phosphate (*β*-TCP) on bone formation around dental implants: a pilot study in sheep. *Clinical Oral Implants Research*.

[47] Hong L, Miyamoto S, Hashimoto N, Tabata Y (2000). Synergistic effect of gelatin microspheres incorporating TGF-*β*1 and a physical barrier for fibrous tissue infiltration on skull bone formation. *Journal of Biomaterials Science, Polymer Edition*.

[48] Hong L, Tabata Y, Miyamoto S (2000). Promoted bone healing at a rabbit skull gap between autologous bone fragment and the surrounding intact bone with biodegradable microspheres containing transforming growth factor-*β*1. *Tissue Engineering*.

[49] Hong L, Tabata Y, Miyamoto S (2000). Bone regeneration at rabbit skull defects treated with transforming growth factor-*β*1 incorporated into hydrogels with different levels of biodegradability. *Journal of Neurosurgery*.

[50] Yamamoto M, Tabata Y, Hong L, Miyamoto S, Hashimoto N, Ikada Y (2000). Bone regeneration by transforming growth factor *β*1 released from a biodegradable hydrogel. *Journal of Controlled Release*.

[51] Furumatsu T, Shen ZN, Kawai A (2003). Vascular endothelial growth factor principally acts as the main angiogenic factor in the early stage of human osteoblastogenesis. *The Journal of Biochemistry*.

[52] Casap N, Venezia NB, Wilensky A, Samuni Y (2008). VEGF facilitates periosteal distraction-induced osteogenesis in rabbits: a micro-computerized tomography study. *Tissue Engineering A*.

[53] Schipani E, Maes C, Carmeliet G, Semenza GL (2009). Regulation of osteogenesis-angiogenesis coupling by HIFs and VEGF. *Journal of Bone and Mineral Research*.

[54] Wang C-J, Huang K-E, Sun Y-C (2011). VEGF modulates angiogenesis and osteogenesis in shockwave-promoted fracture healing in rabbits. *Journal of Surgical Research*.

[55] Tabata Y, Nagano A, Ikada Y, Ikada Y (1999). Biodegradation of hydrogel carrier incorporating fibroblast growth factor. *Tissue Engineering*.

[56] Kanematsu A, Yamamoto S, Ozeki M (2004). Collagenous matrices as release carriers of exogenous growth factors. *Biomaterials*.

[57] Bozlar M, Aslan B, Kalaci A, Baktiroglu L, Yanat AN, Tasci A (2005). Effects of human granulocyte-colony stimulating factor on fracture healing in rats. *Saudi Medical Journal*.

[58] Mifune Y, Matsumoto T, Kawamoto A (2008). Local delivery of granulocyte colony stimulating factor-mobilized CD34-positive progenitor cells using bioscaffold for modality of unhealing bone fracture. *Stem Cells*.

[59] Ishida K, Matsumoto T, Sasaki K (2010). Bone regeneration properties of granulocyte colony-stimulating factor via neovascularization and osteogenesis. *Tissue Engineering A*.

[60] Kuroda R, Matsumoto T, Miwa M (2011). Local transplantation of G-CSF-mobilized CD34^+^ cells in a patient with tibial nonunion: a case report. *Cell Transplantation*.

[61] Lindemann A, Herrmann F, Oster W (1989). Hematologic effects of recombinant human granulocyte colony-stimulating factor in patients with malignancy. *Blood*.

[62] Demetri GD, Griffin JD (1991). Granulocyte colony-stimulating factor and its receptor. *Blood*.

[63] Kojima S, Matsuyama T (1994). Stimulation of granulopoiesis by high-dose recombinant human granulocyte colony-stimulating factor in children with aplastic anemia and very severe neutropenia. *Blood*.

[64] Bishop MR, Tarantolo SR, Geller RB (2000). A randomized, double-blind trial of filgrastim (granulocyte colony- stimulating factor) versus placebo following allogeneic blood stem cell transplantation. *Blood*.

[65] Patte C, Laplanche A, Bertozzi AI (2002). Granulocyte colony-stimulating factor in induction treatment of children with non-Hodgkin’s lymphoma: a randomized study of the French Society of Pediatric Oncology. *Journal of Clinical Oncology*.

[66] Heath JA, Steinherz PG, Altman A (2003). Human granulocyte colony-stimulating factor in children with high-risk acute lymphoblastic leukemia: a Children’s Cancer Group Study. *Journal of Clinical Oncology*.

[67] Wittman B, Horan J, Lyman GH (2006). Prophylactic colony-stimulating factors in children receiving myelosuppressive chemotherapy: a meta-analysis of randomized controlled trials. *Cancer Treatment Reviews*.

[68] Lehrnbecher T, Zimmermann M, Reinhardt D, Dworzak M, Stary J, Creutzig U (2007). Prophylactic human granulocyte colony-stimulating factor after induction therapy in pediatric acute myeloid leukemia. *Blood*.

[69] Inaba H, Cao X, Pounds S (2011). Randomized trial of 2 dosages of prophylactic granulocyte-colony- stimulating factor after induction chemotherapy in pediatric acute myeloid leukemia. *Cancer*.

[70] Luvizuto ER, Tangl S, Zanoni G (2011). The effect of BMP-2 on the osteoconductive properties of *β*-tricalcium phosphate in rat calvaria defects. *Biomaterials*.

[71] Rojbani H, Nyan M, Ohya K, Kasugai S (2011). Evaluation of the osteoconductivity of *α*-tricalcium phosphate, *β*-tricalcium phosphate, and hydroxyapatite combined with or without simvastatin in rat calvarial defect. *Journal of Biomedical Materials Research A*.

[72] Kato E, Lemler J, Sakurai K, Yamada M (2012). Biodegradation property of beta-tricalcium phosphate-collagen composite in accordance with bone formation: a comparative study with bio-oss collagen in a rat critical-size defect model. *Clinical Implant Dentistry and Related Research*.

[73] Zanchetta P, Lagarde N, Uguen A, Marcorelles P (2012). Mixture of hyaluronic acid, chondroitin 6 sulphate and dermatan sulphate used to completely regenerate bone in rat critical size defect model. *Journal of Cranio-Maxillofacial Surgery*.

[74] Tabata Y, Hijikata S, Ikada Y (1994). Enhanced vascularization and tissue granulation by basic fibroblast growth factor impregnated in gelatin hydrogels. *Journal of Controlled Release*.

[75] Levi B, Nelson ER, Brown K (2011). Differences in osteogenic differentiation of adipose-derived stromal cells from murine, canine, and human sources in vitro and in vivo. *Plastic & Reconstructive Surgery*.

[76] Dorozhkin SV (2008). Calcium orthophosphate cements for biomedical application. *Journal of Materials Science*.

[77] Horch H-H, Sader R, Pautke C, Neff A, Deppe H, Kolk A (2006). Synthetic, pure-phase beta-tricalcium phosphate ceramic granules (Cerasorb) for bone regeneration in the reconstructive surgery of the jaws. *International Journal of Oral and Maxillofacial Surgery*.

[78] Weijs WLJ, Siebers TJH, Kuijpers-Jagtman AM, Bergé SJ, Meijer GJ, Borstlap WA (2010). Early secondary closure of alveolar clefts with mandibular symphyseal bone grafts and *β*-tri calcium phosphate (*β*-TCP). *International Journal of Oral and Maxillofacial Surgery*.

[79] Park JH, Choi CG, Jeon SR, Rhim SC, Kim CJ, Roh SW (2011). Radiographic analysis of instrumented posterolateral fusion mass using mixture of local autologous bone and b-TCP (polybone) in a lumbar spinal fusion surgery. *Journal of Korean Neurosurgical Society*.

[80] Jingushi S, Urabe K, Okazaki K (2002). Intramuscular bone induction by human recombinant bone morphogenetic protein-2 with beta-tricalcium phosphate as a carrier: in vivo bone banking for muscle-pedicle autograft. *Journal of Orthopaedic Science*.

[81] Abarrategi A, Moreno-Vicente C, Ramos V, Aranaz I, Sanz Casado JV, López-Lacomba JL (2008). Improvement of porous *β*-TCP scaffolds with rhBMP-2 chitosan carrier film for bone tissue application. *Tissue Engineering A*.

[82] Maus U, Andereya S, Gravius S, Ohnsorge JAK, Niedhart C, Siebert CH (2008). BMP-2 incorporated in a tricalcium phosphate bone substitute enhances bone remodeling in sheep. *Journal of Biomaterials Applications*.

[83] Matsumoto G, Omi Y, Kubota E (2009). Enhanced regeneration of critical bone defects using a biodegradable gelatin sponge and *β*-tricalcium phosphate with bone morphogenetic protein-2. *Journal of Biomaterials Applications*.

[84] Sohier J, Daculsi G, Sourice S, de Groot K, Layrolle P (2010). Porous beta tricalcium phosphate scaffolds used as a BMP-2 delivery system for bone tissue engineering. *Journal of Biomedical Materials Research A*.

[85] Takahashi Y, Yamamoto M, Tabata Y (2005). Osteogenic differentiation of mesenchymal stem cells in biodegradable sponges composed of gelatin and *β*-tricalcium phosphate. *Biomaterials*.

[86] Xu L, Lv K, Zhang W, Zhang X, Jiang X, Zhang F (2012). The healing of critical-size calvarial bone defects in rat with rhPDGF-BB, BMSCs, and *β*-TCP scaffolds. *Journal of Materials Science: Materials in Medicine*.

[87] Matsumoto T, Kawamoto A, Kuroda R (2006). Therapeutic potential of vasculogenesis and osteogenesis promoted by peripheral blood CD34-positive cells for functional bone healing. *The American Journal of Pathology*.

[88] Laing AJ, Dillon JP, Condon ET (2007). Mobilization of endothelial precursor cells: systemic vascular response to musculoskeletal trauma. *Journal of Orthopaedic Research*.

[89] Matsumoto T, Mifune Y, Kawamoto A (2008). Fracture induced mobilization and incorporation of bone marrow-derived endothelial progenitor cells for bone healing. *Journal of Cellular Physiology*.

[90] Janowska-Wleczorek A, Majka M, Ratajczak J, Ratajczak MZ (2001). Autocrine/paracrine mechanisms in human hematopoiesis. *Stem Cells*.

[91] Majka M, Janowska-Wieczorek A, Ratajczak J (2001). Numerous growth factors, cytokines, and chemokines are secreted by human CD34^+^ cells, myeloblasts, erythroblasts, and megakaryoblasts and regulate normal hematopoiesis in an autocrine/paracrine manner. *Blood*.

[92] Chen J-L, Hunt P, Mcelvain M, Black T, Kaufman S, Choi ES-H (1997). Osteoblast precursor cells are found in CD34^+^ cells from human bone marrow. *Stem Cells*.

[93] Iwakura A, Tabata Y, Koyama T (2003). Gelatin sheet incorporating basic fibroblast growth factor enhances sternal healing after harvesting bilateral internal thoracic arteries. *Journal of Thoracic and Cardiovascular Surgery*.

[94] Tabata Y (2003). Tissue regeneration based on growth factor release. *Tissue Engineering*.

[95] Kamitakahara M, Ohtsuki C, Miyazaki T (2008). Review paper: behavior of ceramic biomaterials derived from tricalcium phosphate in physiological condition. *Journal of Biomaterials Applications*.

[96] Ghanaati S, Barbeck M, Detsch R (2012). The chemical composition of synthetic bone substitutes influences tissue reactions in vivo: histological and histomorphometrical analysis of the cellular inflammatory response to hydroxyapatite, beta-tricalcium phosphate and biphasic calcium phosphate ceramics. *Biomedical Materials*.

[97] Liu B, Lun DX (2012). Current application of beta-tricalcium phosphate composites in orthopaedics. *Orthopaedic Surgery*.

[98] Brouard N, Driessen R, Short B, Simmons PJ (2010). G-CSF increases mesenchymal precursor cell numbers in the bone marrow via an indirect mechanism involving osteoclast-mediated bone resorption. *Stem Cell Research*.

[99] Jang J-W, Yun J-H, Lee K-I (2012). Osteoinductive activity of biphasic calcium phosphate with different rhBMP-2 doses in rats. *Oral Surgery, Oral Medicine, Oral Pathology, Oral Radiology and Endodontology*.

[100] Asahara T, Masuda H, Takahashi T (1999). Bone marrow origin of endothelial progenitor cells responsible for postnatal vasculogenesis in physiological and pathological neovascularization. *Circulation Research*.

[101] Taguchi A, Soma T, Tanaka H (2004). Administration of CD34^+^ cells after stroke enhances neurogenesis via angiogenesis in a mouse model. *The Journal of Clinical Investigation*.

[102] Iwasaki H, Kawamoto A, Ishikawa M (2006). Dose-dependent contribution of CD34-positive cell transplantation to concurrent vasculogenesis and cardiomyogenesis for functional regenerative recovery after myocardial infarction. *Circulation*.

[103] Sasaki K, Kuroda R, Ishida K (2008). Enhancement of tendon-bone osteointegration of anterior cruciate ligament graft using granulocyte colony-stimulating factor. *The American Journal of Sports Medicine*.

[104] Lundgren AK, Lundgren D, Hämmerle CHF, Nyman S, Sennerby L (2000). Influence of decortication of the donor bone on guided bone augmentation an experimental study in the rabbit skull bone. *Clinical Oral Implants Research*.

